# Efficacy and safety of rhBMP/β-TCP in alveolar ridge preservation: a multicenter, randomized, open-label, comparative, investigator-blinded clinical trial

**DOI:** 10.1186/s40902-021-00328-0

**Published:** 2021-12-20

**Authors:** Jeong Joon Han, Ah. Ryum Chang, Jaemyung Ahn, Seunggon Jung, Jongrak Hong, Hee-Kyun Oh, Soon Jung Hwang

**Affiliations:** 1 Department of Oral and Maxillofacial Surgery, School of Dentistry, Seoul National University, Seoul, Republic of Korea; 2 Dental Research Institute, Seoul National University, Seoul, Republic of Korea; 3 Department of Oral and Maxillofacial Surgery, Samsung Medical Center, Sungkyunkwan University School of Medicine, Seoul, Republic of Korea; 4 Department of Oral and Maxillofacial Surgery, Dental Science Research Institute, School of Dentistry, Chonnam National University, Gwangju, Republic of Korea; 5 Hwang Soon Jung’s Dental Clinic for Oral and Maxillofacial Surgery, 349, Woonam Building 2, 3F, Gangnam-daero, Seocho-gu, Seoul, 06626 Republic of Korea

**Keywords:** Alveolar ridge preservation, Socket preservation, rhBMP-2; β-TCP, Clinical trial

## Abstract

**Background:**

The aim of this multicenter, randomized, open-label, comparative, investigator-blinded study was to investigate the efficacy and safety of recombinant human bone morphogenetic protein 2 (rhBMP-2) combined with β-TCP (rhBMP-2/β-TCP) in alveolar ridge preservation.

**Materials and methods:**

Eighty-four subjects from three centers were enrolled in this clinical trial. After tooth extraction, rhBMP-2/β-TCP (*n* = 41, test group) or β-TCP (*n* = 43, control group) were grafted to the extraction socket with an absorbable barrier membrane for alveolar ridge preservation. Using computed tomography images obtained immediately after and 12 weeks after surgery, changes in the alveolar bone height and width were analyzed for each group and compared between the two groups.

**Results:**

Both the test and control groups showed a significant decrease in alveolar bone height in the 12 weeks after surgery (both groups, *p* < 0.0001). However, the test group exhibited a significantly lower decrease in alveolar bone height than the control group (*p* = 0.0004). Alveolar bone width also showed significantly less resorption in the test group than in the control group for all extraction socket levels (ESL) (*p* = 0.0152 for 75% ESL; *p* < 0.0001 for 50% ESL; *p* < 0.0001 for 25% ESL). There were no statistically significant differences in the incidence of adverse events between the two groups. No severe adverse events occurred in either group.

**Conclusions:**

The results of this study suggest that rhBMP-2/β-TCP is a safe graft material that provides a high alveolar bone preservation effect in patients receiving dental extraction.

**Trial registration:**

Clinicaltrials.gov, NCT02714829, Registered 22 March 2016

## Background

The alveolar ridge undergoes normal physiological bone resorption after tooth extraction. Several studies have demonstrated that the major bony changes around the extraction socket occurred during the 12 months after extraction, where two-thirds of total resorption in the alveolar bone width and almost all resorption of alveolar bone height occurred within the first 3 months after tooth extraction [1, 2]. In a systematic review by Van der Weijden et al. [3], the mean amount of alveolar resorption during the post-extraction healing period was 3.87 mm for width and 1.67–2.03 mm for height. Insufficient height and width of the residual alveolar bone make it difficult to place the implant in an ideal position during subsequent rehabilitation with dental implants and requires complicated bone grafting. The prosthesis is also esthetically compromised.

To minimize alveolar bone resorption following tooth extraction and maintain favorable volume and morphology of the alveolar ridge for future restoration, various alveolar ridge preservation (ARP) techniques have been developed and investigated. As biomaterials for filling extraction sockets, bone substitutes, such as demineralized freeze-dried bone, deproteinized bovine bone mineral, hydroxyapatite (HA), and beta-tricalcium phosphate (β-TCP), have been most commonly used, and the grafted materials in the extraction socket are usually covered with resorbable or non-resorbable barrier membrane [4–8]. As an alternative to bone grafting, several investigators have used absorbable collagen sponge (ACS) for the extraction socket with various growth factors and reported comparable treatment outcomes to bone grafts [9, 10]. In terms of wound closure after application of the graft material in the extraction socket, the primary closure method through releasing incision and advancement of the mucoperiosteal flap or open wound method with placing barrier membrane for coverage of grafted material without flap elevation have been used. In a recent systematic review with meta-analysis, Avila-Ortiz et al. [11] reported that ARP could reduce vertical and horizontal bone resorption following tooth extraction by 1.72 mm and 1.99 mm, respectively.

β-TCP, one of the most widely used alloplastic bone graft materials along with HA, has been used for bone regeneration and augmentation in the oral and maxillofacial region due to its biocompatibility, biodegradability, and osteoconductivity [12]. β-TCP has the property of being replaced with new bone through bone resorption and remodeling processes after grafting [13]. In addition, clinical efficacy and safety have been proven in many animal studies and clinical trials in the fields of orthopedic surgery and dentistry. However, due to the lack of growth factors and cellular components, β-TCP does not have osteoinductive properties [6]. Therefore, several bioactive molecules have been combined with β-TCP to enhance bone regeneration.

Recently, many efforts have been made to improve bone regeneration by combining various growth factors with bone substitutes [14]. Bone morphogenetic protein (BMP), which was discovered by Urist in 1965 [15], has been extensively investigated in the field of bone regeneration as an effective enhancer of osteogenesis. BMP is a superfamily of transforming growth factor-β (TGF-β), and it has high osteoinductive capacity resulting from chemotaxis, proliferation, and differentiation of mesenchymal stem cells [16–19]. Among various recombinant human BMPs (rhBMPs), rhBMP-2 has been used together with several bone substitutes to enhance bone regeneration due to its high osteogenic capacity. Jung et al. [17] used rhBMP-2 with a xenogenic bone substitute for guided bone regeneration in the atrophic edentulous area and reported the potential of BMP-2 to enhance bone regeneration treatment. Kim et al. [18] used rhBMP-2 with hydroxyapatite for maxillary sinus floor augmentation and reported good efficacy in early bone formation.

To our knowledge, there are few reports on the changes in alveolar bone height and width after ARP using rhBMP-2 combined with β-TCP (rhBMP-2/β-TCP). Thus, in this clinical trial, we investigated the efficacy and safety of rhBMP-2/β-TCP in ARP, compared to β-TCP.

## Materials and methods

### Clinical trial design

This clinical trial was designed as a 12-week multicenter, randomized, open-label, comparative, investigator-blinded study conducted in three centers (Seoul National University Dental Hospital, Samsung Medical Center, and Chonnam National University), from April 2016 to September 2017. It was registered at ClinicalTrials.gov (ID: NCT02714829) and approved by the Institutional Review Board of the three centers (approval no. CDE16001 in Seoul National University Dental Hospital, 2016-01-035 at Samsung Medical Center, CNUH-2016-140 in Chonnam National University Hospital) and Ministry of Food and Drug Safety of Korea (approval no.: 472).

**Fig. 1:**
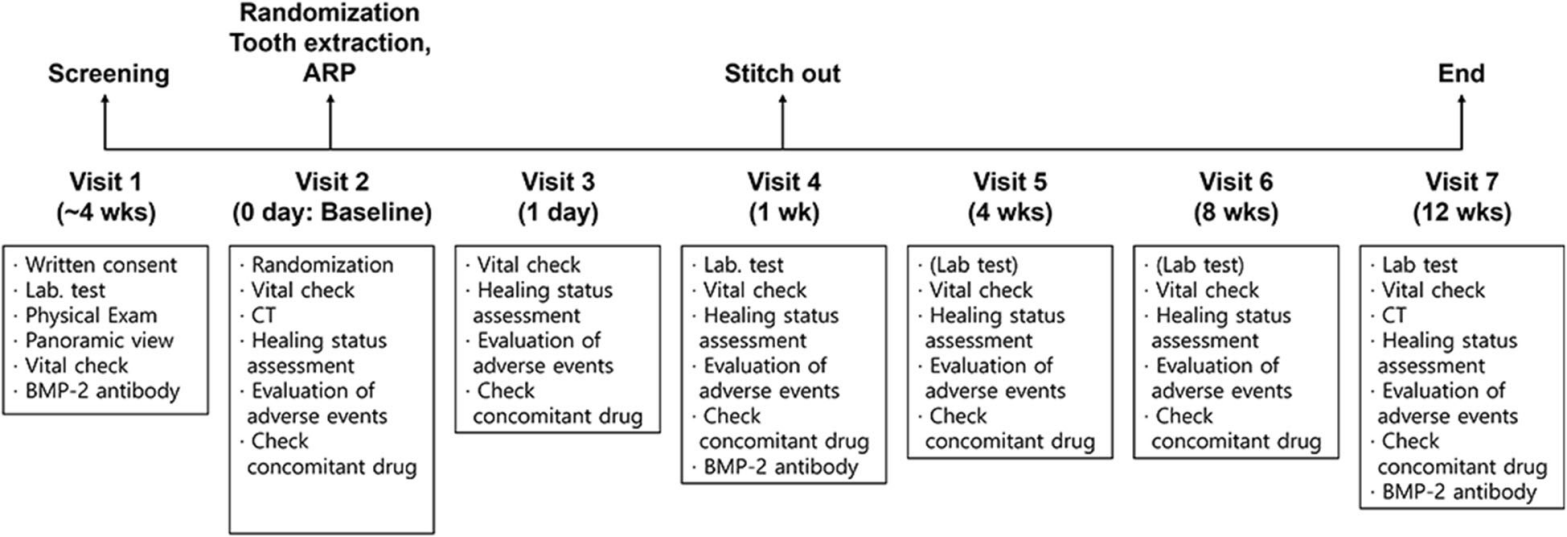
Schematic diagram of the protocol in the clinical trial

The protocol included seven visits per subject, and the flow diagram is shown in Fig. 1. At the first visit (screening), candidates were assessed using the following examinations: demographic survey, clinical examinations, physical examination with vital sign checks, medical history, radiographic examination using panoramic radiograph, laboratory test including common blood cell count, blood coagulation test, serum biochemical test, urine analysis, and immune response test for BMP-2. The subjects were selected according to the inclusion and exclusion criteria. At the second visit, tooth extraction and ARP were performed after the subjects were randomized into the test and control groups, and multi-detector computed tomography (CT) was performed to determine the baseline immediately after surgery. The sutures were removed 1 week after the surgery (visit 4). The subjects were observed for 12 weeks following surgery to evaluate their efficacy and safety. Twelve weeks after surgery, CT was performed to evaluate the changes in the alveolar ridge.

### Grafted materials and subjects

**Fig. 2:**
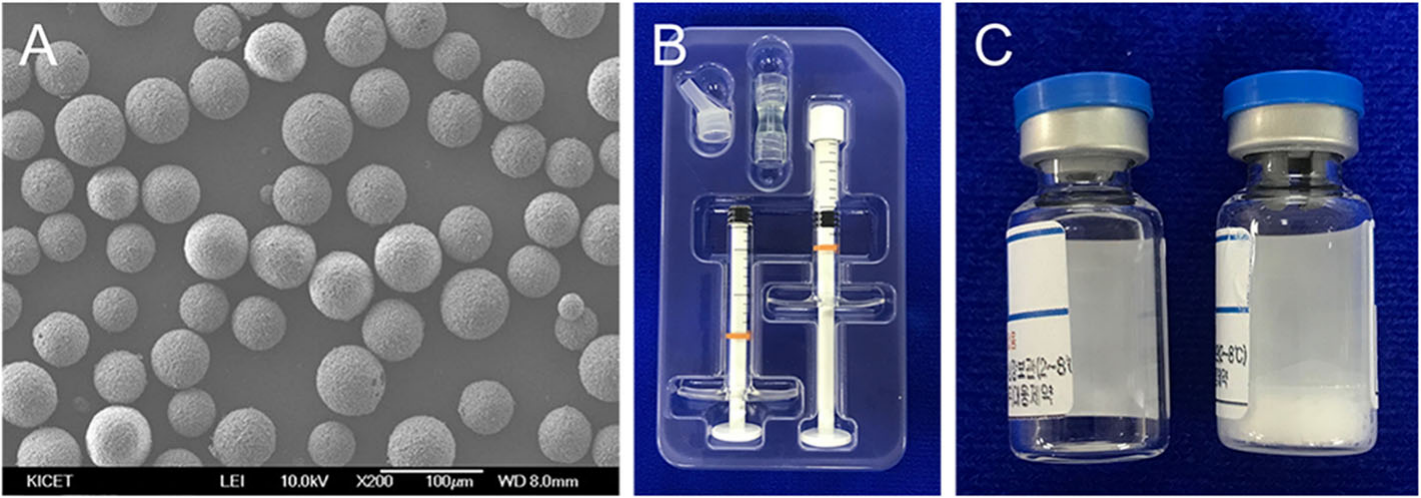
rhBMP-2/β-TCP used in this study. **A** Field emission scanning electron microscope image of β-TCP (x 200) **B** Carrier part including syringe containing 1.5 g of β-TCP, empty, unused syringe, connector and syringe tip **C** recombinant human BMP-2 part including E. coli-derived rhBMP-2 powder and water for injection

The graft materials used were rhBMP-2/β-TCP (Novosis Inject Dent, CGBio, Gyeonggi-do, Korea) for the test group and β-TCP (Excelos Inject, CGbio, Gyeonggi-do, Korea) for the control group (Fig. 2). The rhBMP-2/β-TCP used in this study consisted of a carrier and rhBMP-2. The carrier was composed of β-TCP microspheres (diameter, 45–75 μm; porosity, higher than 65 vol.%) with Poloxamer 407 based hydrogel composite and was sterilized with gamma radiation. E. coli-derived rhBMP-2, provided as a lyophilized powder, was added to the carrier in a 2.5 mg/mL rhBMP-2 solution. After 0.5 mg rhBMP-2 was dissolved with 0.2 mL of water, 0.2 mL of rhBMP-2 solution was moved to an empty syringe. The syringe, including 0.2 mL of rhBMP-2 solution, was connected to a syringe containing a 1.5-g carrier using a connector. The contents were mixed by pushing both syringe rods at least ten times and were gathered into one syringe. After detachment of the connector and attachment of the syringe tip, rhBMP-2/β-TCP was grafted into the extraction socket up to the level of the alveolar ridge crest. For the control group, β-TCP, which had the same composition and properties as the carrier used in rhBMP-2/β-TCP, was used.

To be included in this study, subjects needed to meet the following inclusion criteria: (1) patients aged 19 to 80 years who required dental extraction, (2) residual alveolar bone surrounding the tooth: 50% and above of the original alveolar bone height (4 mm and above), (3) no evidence of severe periodontitis, and (4) patients who voluntarily consented to clinical trials and can follow the protocols of the clinical trial. Patients who had already undergone tooth extraction could participate if the extraction was performed within 3 days. The following exclusion criteria were applied: (1) for extraction of the third molar, (2) patients with disease that requires continuous prophylactic antibiotics, (3) patients with major systemic disease, (4) patients who require long-term steroid administration, (5) females who are pregnant or have childbearing potential, (6) patients with severe periodontitis and vertical alveolar bone resorption more than 50%, (7) inadequate oral hygiene, (8) patients who have participated in other clinical trials and received drug treatment or treatment using other medical device within the past 90 days, (9) immune disease including acquired immune deficiency syndrome, (10) patients who recently received periodontal surgery in the target tooth of clinical trial (within 2 months in cases of partial healing such as gingivectomy, periodontal flap surgery, and within 6 months in procedures associated with dental extraction such as guided periodontal tissue regeneration, and guided alveolar bone regeneration), (11) patients who exhibit hypersensitivity to the component included in the grafted materials used in this clinical trial, (12) alcohol use disorder and substance abuse disorder, (13) other patients determined inappropriate for the clinical trial.

### Determination of the sample size

The sample size was determined based on previous studies conducted to compare the two groups according to the inclusion of BMP-2 (weighted mean difference, − 0.72 mm; standard deviation, 1.10 mm) [20, 21]. A minimum of 74 subjects (37 subjects in each group) were required to detect a difference between the two groups with 80% power and an alpha value of 0.05. Finally, assuming a dropout rate and serious protocol violation rate of 15%, the total required number of enrolled subjects was 88 (44 subjects in each group).

### Randomization and blinding

Subjects were randomly assigned to the test or control groups with a 1:1 allocation ratio by the randomization code, which was generated using the SAS PROC PLAN procedure (SAS Institute Inc., Cary, NC, USA), using a stratified block randomization method. An individual randomization envelope was prepared and sent to the principal investigator of each clinical trial center. The investigator could only know the assigned group after the registration of the subject was completed.

Although the blinding was not applied to the subjects and investigators in this clinical trial, two external independent evaluators were blinded to the group subjects when evaluating the CT data after the end of the clinical trial.

### Surgical procedures

**Fig. 3:**
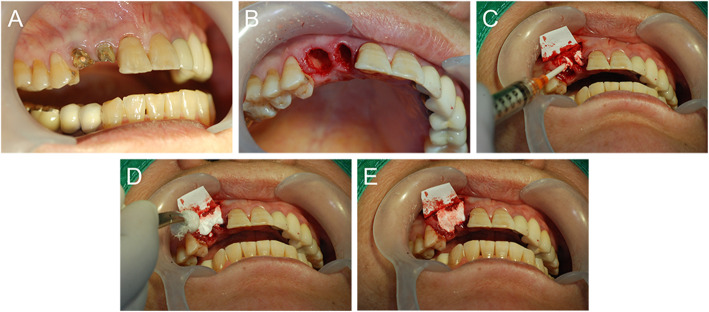
Surgical procedures performed in this study. **A** Initial clinical photograph. **B** After extraction. **C**, **D**, and **E** Application of the grafted material into the extraction socket up to the crestal level of the remaining alveolar bone (rhBMP-2/ β-TCP for test group and β-TCP for control group)

The surgical procedure was performed during the second visit (Fig. 3). After local anesthesia, tooth extraction was performed carefully with minimal damage to the surrounding alveolar bone, and the remaining granulation tissue and infected tissue were removed. For each group, the allocated graft material was applied to the extraction socket up to the crestal level of the remaining alveolar bone. The amount of grafted rhBMP-2/β-TCP or β-TCP can be different depending on the size of each extraction socket, but the concentration of rhBMP-2 in the grafted β-TCP was kept the same by making the mixing ratio the same before grafting. After the absorbable barrier membrane (Remaix, Matricel GmbH, Herzogenrath, Germany) was placed on the extraction socket to cover the graft material, wound closure was performed with 4-0 Vicryl (Ethicon, Somerville, NJ, USA) at the level of approximation of the buccal and lingual/palatal flaps.

### Efficacy outcome

**Fig. 4:**
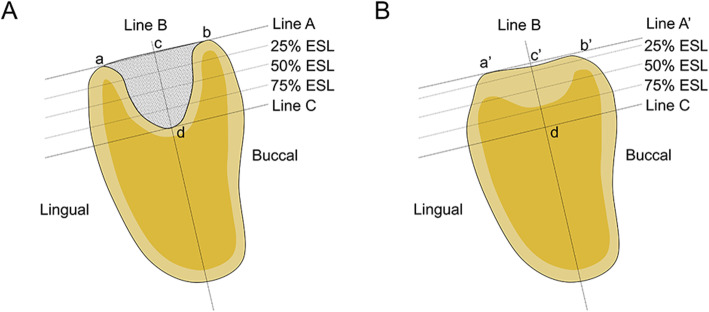
Landmarks and linear measurement parameters used to evaluate changes in the alveolar bone height and width in this study (**A**, immediately after surgery; **B**, 6 months after ARP). Line A was the line passing through points a and b, where points a and b were defined as the most superior point of the lingual and buccal alveolar bone, respectively. Point c was the point that bisects line A, and point d was the apex of the extraction socket. The line passing through points c and d was defined as line B. Line C was set as the line perpendicular to line B and passing through point d. The height of the alveolar bone immediately after surgery was measured as the distance from point c to point d. The width of the alveolar bone was measured at the points where the alveolar bone height was divided into quarters. To measure the changes of the alveolar ridge 12 weeks after surgery, the reference lines B and C, reference lines for 25%, 50%, and 75% ESL, and point d were transferred to the CT images taken 12 weeks after surgery. Point a’ and b’ were marked as the most superior point of the lingual and buccal alveolar bone, and line A’ was defined as the line passing through points a’ and b’. Point c’ was the intersection of line A’ and line B. By measuring the distance from point c’ to point d, the alveolar bone height at 12 weeks after surgery was measured. The width of the alveolar bone was measured at the points where the alveolar bone height was divided into quarters

In this study, the primary efficacy outcome was the changes in the alveolar bone height compared with the baseline at 12 weeks after tooth extraction and ARP, and the secondary efficacy outcome was the changes in the alveolar bone width compared with the baseline at 12 weeks after tooth extraction and ARP. The alveolar bone width was measured at 75%, 50%, and 25% of the extraction socket length (ESL). CT data taken at baseline and 12 weeks after surgery were superimposed based on stable anatomic structures that did not change during the follow-up period using OnDemand3D software (Cybermed, Seoul, Korea). The measurement method was determined based on previous studies that analyzed the changes in the alveolar bone after ARP using CT data [10, 20, 22]. Landmarks and linear measurement parameters used to evaluate changes in the alveolar bone height and width in this study are shown in Fig. 4. Line A was the line passing through points a and b, where points a and b were defined as the most superior point of the lingual and buccal alveolar bone, respectively. Point c was the point that bisects line A, and point d was the apex of the extraction socket. The line passing through points c and d was defined as line B. Line C was set as the line perpendicular to line B and passing through point d. The height of the alveolar bone immediately after surgery was measured as the distance from point c to point d. The width of the alveolar bone was measured at the points where the alveolar bone height was divided into quarters. To measure the changes of the alveolar ridge 12 weeks after surgery, the reference lines B and C, reference lines for 25%, 50%, and 75% ESL, and point d were transferred to the CT images taken 12 weeks after surgery. Point a’ and b’ were marked as the most superior point of the lingual and buccal alveolar bone, and line A’ was defined as the line passing through points a’ and b’. Point c’ was the intersection of line A’ and line B. By measuring the distance from point c’ to point d, the alveolar bone height at 12 weeks after surgery was measured. The width of the alveolar bone was measured at the points where the alveolar bone height was divided into quarters.

### Safety assessment

Adverse events were collected from visit 2 to the end of the clinical trial. When an adverse event occurred, the symptoms, onset date, resolution date, degree of severity, and suspected causal relationship with the grafted material were recorded. All adverse events were classified as preferred term by system organ class according to the MedDRA (Medical Dictionary of Regulatory Activities). The degree of severity was divided into three levels: mild, moderate, and severe. Mild referred to symptoms that caused minimal discomfort and were easily tolerated without interfering with normal daily life, and moderate referred to symptoms that significantly interfered with normal daily life. Severe referred to symptoms that made normal daily life impossible. The causal relationship was evaluated considering the following factors: (1) evidence that the graft material was used, (2) time sequence of use of the graft material and occurrence of adverse events, (3) cases that were most likely to be explained by the use of the graft material than other causes, (4) resolution of the symptoms of the adverse event after removal of the graft material.

To assess the safety of the application of the graft material, clinical examinations, vital signs, and laboratory tests, including common blood cell count, blood coagulation test, serum biochemical test, and urine analysis, were performed with the immune response test of BMP-2 and monitoring of adverse events. Vital signs, including the systolic and diastolic blood pressure, pulse rate, and body temperature were measured at each visit prior to other scheduled tests. Immune response tests for BMP-2 were performed at visits 1, 4, and 7. At visits 4 and 7, evaluations were only conducted for the test group. The BMP-2-antibody in the serum was analyzed by enzyme-linked immunosorbent assay with purified rabbit anti-human BMP-2 (RHF913; Antigenix America, NY, USA). Healing status was assessed using a visual analog scale for postoperative pain, healing grade evaluated by the investigator, and white blood cell count. When measuring the visual analogue scale, the subject evaluated the degree of pain in the range of 0 to 10, and it was judged that the higher the value, the more severe the pain. The healing grade was evaluated by dividing the healing degree of the surgical site into 5 grades from visit 4 to visit 7: grade 0, absence of inflammation; grade 1, mild inflammation (partial involvement); grade 2, mild inflammation (entire involvement); grade 3, moderate inflammation; grade 4, severe inflammation [23]. White blood count was checked at visit 4 and visit 7 for objective evaluation of postoperative infection.

### Statistical analysis

Statistical analysis was performed using SAS software (SAS Institute, Cary, NC, USA). In the comparison of demographic data between the two groups, the two-sample *t* test or Wilcoxon’s rank-sum test was performed for continuous variables, and the chi-square test or Fisher’s exact test was conducted for categorical variables. When a missing value occurred in the analysis, it was treated as missing without correction.

To compare the change in alveolar bone height at 12 weeks after tooth extraction and ARP between the test and control groups, an analysis of covariance (ANCOVA), which adjusted the baseline as well as the trial center (a stratification factor), was conducted. When the lower limit of the 95% two-sided confidence interval for the differences between the test and control groups estimated in this model was greater than 0, the test group was determined to be superior to the control group. Additionally, a paired *t* test or Wilcoxon’s signed-rank test was conducted to evaluate whether the decrease in alveolar bone height in each group was statistically significant. Least-square mean (LSM), which was corrected for the baseline and institution, standard error, two-sided 95% confidence interval corresponding to the LSM difference in contrast to the control group, and the *p* value were presented for each group.

To evaluate whether the decrease in alveolar bone width at 75%, 50%, and 25% ESL in each group at 12 weeks after surgery was statistically significant, a paired *t* test or Wilcoxon’s signed-rank test was conducted, and ANCOVA analysis, which adjusts the baseline as well as the trial center (a stratification factor) was conducted to evaluate the significance of the difference between groups. Additionally, LSM, standard error, two-sided 95% confidence interval corresponding to the LSM difference in contrast to the control group, and the *p* values were presented for each group.

Safety assessment was conducted on all subjects who underwent tooth extraction and the application of the graft material. Subjects enrolled in this clinical trial but dropped out before the extraction and application of the graft material were excluded from the safety assessment. The ratio of subjects who experienced adverse events and the corresponding 95% confidence interval was presented for each treatment group. To test whether there was a difference in the incidence rate of adverse events between the two groups, Fisher’s exact test was conducted. All adverse events were organized according to severity and relationship with the graft material. For the continuous variables in the laboratory test, vital signs, and healing status assessment, differences between two visits were tested using a paired *t* test or Wilcoxon’s signed-rank test, and comparisons between the two groups were evaluated using a two-sample test or Wilcoxon’s rank-sum test. In terms of the categorical variables, McNemar’s test was conducted to evaluate the differences between two visits for each group, and the chi-square test or Fisher’s exact test was conducted to compare the differences between the groups.

## Results

**Fig. 5:**
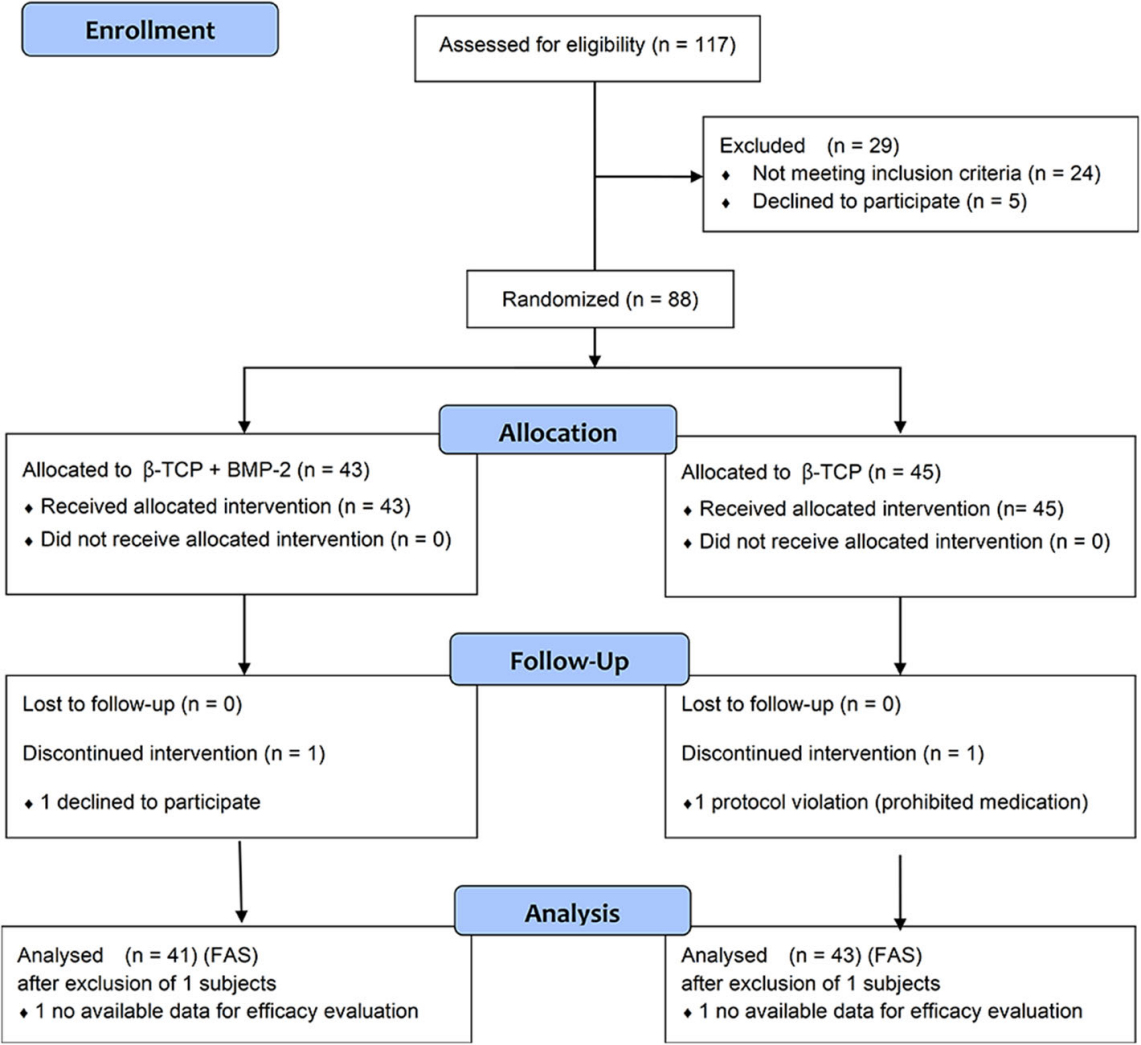
Flow diagram of a multicenter, randomized, open-label, comparative, investigator-blinded clinical trial of rhBMP-2/β-TCP (test group) for alveolar ridge preservation in alveolar ridge preservation compared with β-TCP (control group)

The flow diagram of this clinical trial is presented in Fig. 5. Among 117 candidates from three trial centers who received screening after written consent, 29 candidates (24 candidates incompatible in the inclusion/exclusion criteria and five candidates whose legally acceptable representatives or candidates themselves requested discontinuation in participating in the clinical trial) were excluded. Of the 88 randomized subjects (test group, 43 subjects; control group, 45 subjects), 86 subjects (test group, 42 subjects; control group, 44 subjects) completed this clinical trial, and a total of two subjects dropped out during the trial for consent withdrawal (one test group) and for accompanying administration of surgery, drugs, or medical device that may affect the safety and efficacy evaluation (one control group). Of the 86 subjects who completed the clinical trial, 84 subjects were included in the full analysis set (FAS) because two subjects (one each in the test and control groups) had insufficient data for analyzing the efficacy. The per-protocol set (PPS) was the same as FAS.

In the FAS, the test and control groups had 41 subjects (male:female = 26:15; mean age, 56.61 ± 14.57; age range, 19 to 74 years) and 43 subjects (male:female = 31:12; mean age, 57.51 ± 12.04 years; age range, 23 to 75 years), respectively (Table 1). Twelve subjects were fertile women who had a negative pregnancy test. The positions of the teeth enrolled in the clinical trial for each subject were the right maxillary teeth, left maxillary teeth, right mandibular teeth, and left mandibular teeth in 28 (34.57%), 26 (32.10%), 14 (17.28%), and 13 subjects (16.05%), respectively. There were no significant differences between the two groups in terms of age, sex, height, body weight, body mass index, smoking status, and tooth position.

**Table 1:** Demographic and baseline characteristics (full analysis set)

	Test group (*N* = 41)	Control group (*N* = 43)	Total (*N* = 84)
Age, years			
Mean	56.61	57.51	57.07
SD	14.57	12.04	13.26
Sex, *n* (%)			
Male	26 (63.41)	31 (72.09)	57 (67.86)
Female	15 (36.59)	12 (27.91)	27 (32.14)
Height, cm			
Mean	164.15	166.40	165.30
SD	8.93	9.11	9.04
Body weight, kg			
Mean	66.23	69.19	67.74
SD	11.70	10.00	10.90
BMI, kg/m^2^			
Mean	24.45	24.95	24.71
SD	2.92	2.80	2.86
Smoking, *n* (%)			
Yes	8 (19.51)	7 (16.28)	15 (17.86)
No	28 (68.29)	29 (67.44)	57 (67.86)
Former smoker	5 (12.20)	7 (16.28)	12 (14.29)
Cigarettes/day, *n*			
Mean	17.08	15.55	16.29
SD	11.16	12.12	11.47
Position of the enrolled tooth			
Right maxilla	15 (38.46)	13 (30.95)	28 (34.57)
Left maxilla	11 (28.21)	15 (35.71)	26 (32.10)
Right mandible	7 (17.95)	6 (14.29)	13 (16.05)
Left mandible	6 (15.38)	8 (19.05)	14 (17.28)

*p* value for each variable: not significant. Wilcoxon’s rank-sum test: age, BMI, cigarettes/day; chi-square test: sex, smoking, position of the enrolled tooth; two sample *t* test: height, body weight

### Efficacy outcome

**Fig. 6:**
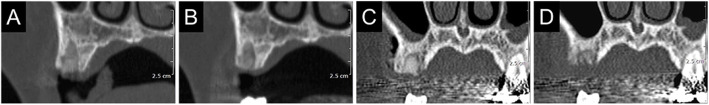
Computed tomography images for efficacy assessment. Test group (**A**, immediately postoperative; **B**, 12 weeks after surgery) showed a better alveolar ridge preservation effect at 12 weeks after surgery than the control group (**C**, immediately postoperative; **D**, 12 weeks after surgery)

The mean changes in alveolar bone height at 12 weeks after ARP compared to the baseline were − 0.51 ± 0.94 mm (*p* < 0.0001) in the test group, and − 1.50 ± 1.26 mm (*p* < 0.0001) in the control group (comparison between the two groups, *p* < 0.0001) (Table 2) (Fig. 6). The LSM, which was corrected for the baseline and institution, was − 0.50 ± 0.20 mm in the test group and − 1.42 ± 0.19 mm in the control group. The test group exhibited a lesser decrease in alveolar bone height than the control group, and the difference between the two groups was a statistically significant difference of 0.92 ± 0.25 mm (*p* = 0.0004). The lower limit of the 95% two-sided confidence interval was 0.43 mm, confirming that the test group had a superior effect on ridge preservation compared to the control group.

**Table 2:** Changes in alveolar bone height (mm) at week 12

	Test group (*N* = 41)	Control group (*N* = 43)
*Change from baseline at week 12*		
Mean ± SD	− 0.51 ± 0.94	− 1.50 ± 1.26
LS mean ± SE	− 0.50 ± 0.20	− 1.42 ± 0.19
Median	− 0.17	− 1.32
Min, max	− 4.52, 0.44	− 5.63, 0.25
*p* value^*^	< 0.0001 (*w*)	< 0.0001 (*w*)
*LS mean difference (SE) with control group*	0.92 (0.25)	
95% CI for the LS mean difference	[0.43, 1.41]	
*p* value^†^	0.0004	

*SD* standard deviation, *LS mean* least-square mean, *SE* standard error, *CI* confidence interval

^*^Testing for within-treatment group (paired *t* test (*t*) or Wilcoxon’s signed-rank test (*w*))

^†^Testing for between-treatment group (ANCOVA model with baseline value and trial center as covariates)

The mean changes in alveolar bone width at 12 weeks after ARP compared to the baseline were − 0.33 ± 0.46 mm (*p* < 0.0001), and − 0.78 ± 1.12 mm (*p* < 0.0001) at 75% ESL, − 0.33 ± 0.53 mm (*p* < 0.0001) and − 1.81 ± 2.05 mm (*p* < 0.0001) at 50% ESL, and − 1.08 ± 2.11 mm (*p* < 0.0001) and − 4.13 ± 3.56 mm (*p* < 0.0001) at 25% ESL in the test and control groups, respectively (Tables 3, 4, and 5). The LSM was − 0.23 ± 0.15 mm, and − 0.70 ± 0.15 mm at 75% ESL, − 0.19 ± 0.26 mm and − 1.69 ± 0.25 mm at 50% ESL, and − 0.93 ± 0.50 mm and − 3.96 ± 0.48 mm at 25% ESL in the test and control groups, respectively. The test group exhibited a significantly lower decrease in alveolar bone width than the control group for all ESLs (*p* = 0.0152 for 75% ESL; *p* < 0.0001 for 50% ESL; *p* < 0.0001 for 25% ESL).

**Table 3:** Changes in alveolar bone width for 75% extraction socket length (mm) at week 12

	Test group (*N* = 41)	Control group (*N* = 43)
*Change from baseline at week 12*		
Mean ± SD	− 0.33 ± 0.46	− 0.78 ± 1.12
LS mean ± SE	− 0.23 ± 0.15	− 0.70 ± 0.15
Median	− 0.08	− 0.32
Min, max	− 1.97, 0.00	− 5.22, 0.00
*p* value^*^	< 0.0001 (w)	< 0.0001 (w)
*LS mean difference (SE) with control group*	0.46 (0.19)	
95% C.I for the LS mean difference	[0.09, 0.84]	
*p* value^†^	0.0152	

*SD* standard deviation, *LS mean* least-square mean, *SE* standard error, *CI* confidence interval

^*^Testing for within-treatment group (paired *t* test (*t*) or Wilcoxon’s signed-rank test (*w*))

^†^Testing for between-treatment group (ANCOVA model with baseline value and trial center as covariates)

**Table 4:** Changes in alveolar bone width for 50% extraction socket length (mm) at week 12

	Test group (*N* = 41)	Control group (*N* = 43)
*Change from baseline at week 12*		
Mean ± SD	− 0.33 ± 0.53	− 1.81 ± 2.05
LS mean ± SE	− 0.19 ± 0.26	− 1.69 ± 0.25
Median	− 0.05	− 1.29
Min, max	− 2.02, 0.84	− 10.51, 0.00
*p* value^*^	< 0.0001 (w)	< 0.0001 (w)
*LS mean difference (SE) with control group*	1.50 (0.32)	
95% CI for the LS mean difference	[0.86, 2.14]	
*p* value^†^	< 0.0001	

*SD* standard deviation, *LS mean* least-square mean, *SE* standard error, CI confidence interval

^*^Testing for within-treatment group (paired *t* test (*t*) or Wilcoxon’s signed-rank test (*w*))

^†^Testing for between-treatment group (ANCOVA model with baseline value and trial center as covariates).

**Table 5:** Changes in alveolar bone width for 25% extraction socket length (mm) at week 12

	Test group (*N* = 41)	Control group (*N* = 43)
*Change from baseline at week 12*		
Mean ± SD	− 1.08 ± 2.11	− 4.13 ± 3.56
LS Mean ± SE	− 0.93 ± 0.50	− 3.96 ± 0.48
Median	− 0.28	− 3.04
Min, max	− 9.87, 0.00	− 13.84, 0.00
*p* value^*^	< 0.0001 (*w*)	< 0.0001 (*w*)
*LS mean difference (SE) with control group*	3.03 (0.61)	
95% CI for the LS mean difference	[1.81, 4.24]	
*p* value^†^	< 0.0001	

*SD* standard deviation, *LS mean* least-square mean, *SE* standard error, *CI* confidence interval

^*^Testing for within-treatment group (paired *t* test (*t*) or Wilcoxon’s signed-rank test (*w*))

^†^Testing for between-treatment group (ANCOVA model with baseline value and trial center as covariates)

### Assessment of safety

A safety assessment was conducted for 88 subjects who received ARP after tooth extraction (Table 6). Thirteen treatment-emergent adverse events (adverse events after the application of the graft material) were observed in nine of 88 subjects (10.23%). For each group, there were two subjects in the test group (4.65%, two cases) and seven subjects in the control group (15.56%, 11 cases), and the difference between the two groups in the incidence of adverse events was not statistically significant (*p* = 0.1577). With regard to the relationship between adverse events and graft material, none of the cases in the test group exhibited relationships. In the control group, ten cases exhibited no relationship, while one case with mild severity (toothache) was reported to be probably related to the graft material and was resolved with medication. There were no severe treatment-emergent adverse events related to the graft material. The incidence of adverse events by systemic organ class and preferred term is also presented in Table 7.

**Table 6:** Summary of adverse events in this clinical trial

	Test group (*N* = 43)	Control group (*N* = 45)	Total (*N* = 48)
*Patients with adverse events*	2 (4.65)	7 (15.56)	9 (10.23)
95% CI	0.57, 15.81	6.49, 29.46	4.78, 18.53
*p* value*			0.1577
*Severity of cases with adverse events*			
Mild	2	9	11
Moderate	0	2	2
Severe	0	0	0
*Relationship to the grafted material*			
Definitely related	0	0	0
Probably related	0	1	1
Possibly related	0	0	0
Possibly not related	0	0	0
Definitely not related	2	10	12
Unknown	0	0	0

^*^Fisher’s exact test

Patients with adverse events are presented as “number of patients (percentage of patients) [number of events]” and others are presented as “number of events”

**Table 7:** Incidence of adverse events by systemic organ class and preferred term

	Test group (*N* = 43)	Control group (*N* = 45)	Total (*N* = 88)
*Patients with adverse events*	2 (4.65) [2]	7 (15.56) [11]	9 (10.23) [13]
*Gastrointestinal disorders*	1 (2.33) [1]	4 (8.89) [4]	5 (5.68) [5]
Toothache	0	2 (4.44) [2]	2 (2.27) [2]
Gingival bleeding	1 (2.33) [1]	0	1 (1.14) [1]
Gingival pain	0	1 (2.22) [1]	1 (1.14) [1]
Sensitivity of teeth	0	1 (2.22) [1]	1 (1.14) [1]
*Nervous system disorders*	0	2 (4.44) [2]	2 (2.27) [2]
Hepatic encephalopathy	0	1 (2.22) [1]	1 (1.14) [1]
Lumbosacral radiculopathy	0	1 (2.22) [1]	1 (1.14) [1]
*Metabolism and nutrition disorders*	0	1 (2.22) [1]	1 (1.14) [2]
Hypoalbuminemia	0	1 (2.22) [1]	1 (1.14) [1]
Hypomagnesaemia	0	1 (2.22) [1]	1 (1.14) [1]
*Infections and infestations*	0	1 (2.22) [1]	1 (1.14) [1]
Periodontitis	0	1 (2.22) [1]	1 (1.14) [1]
*Investigations*	0	1 (2.22) [1]	1 (1.14) [1]
Blood glucose increased	0	1 (2.22) [1]	1 (1.14) [1]
*Skin and subcutaneous tissue disorders*	1 (2.33) [1]	0	1 (1.14) [1]
Pruritus	1 (2.33) [1]	0	1 (1.14) [1]

Patients with adverse events are presented as “number of patients (percentage of patients) [number of events]” and others are presented as “number of events”

With respect to vital signs, including blood pressure, pulse, and body temperature, all changes in vital signs within the group were within the normal range, and there were no significant differences between the two groups (systolic pressure: *p* = 0.6306, *p* = 0.4940, *p* = 0.9695, *p* = 0.1894, and *p* = 0.6852 at visits 3, 4, 5, 6, and 7, respectively; diastolic pressure: *p =* 0.1840, *p* = 0.4635; *p* = 0.1283; *p* = 0.5605, and *p* = 0.2598 at visits 3, 4, 5, 6, and 7, respectively; pulse: *p* = 0.7402, *p* = 0.2931, *p* = 0.5072, *p* = 0.2430, and *p* = 0.1946 at visits 3, 4, 5, 6, and 7, respectively; body temperature, *p* = 0.8051, *p* = 0.1767, *p* = 0.3616, *p* = 0.0595; *p* = 0.2527 at visits 3, 4, 5, 6, and 7, respectively). Healing status was assessed using the visual analogue scale, degree of healing grade, and white blood cell value, and there was no statistically significant difference between the two groups in visual analogue scale and white blood cell value (visual analogue scale: *p* = 0.4512, *p* = 0.7578, *p* = 0.7374, *p* = 0.4057, and *p* = 0.5297 at visits 3, 4, 5, 6, and 7, respectively; white blood cell value: *p* = 0.7502 and *p* = 0.1560 at visits 4 and 7, respectively). The degree of healing grade was evaluated at visits 4, 5, 6, and 7. At all visits, no inflammation was observed in all subjects, which was rated as grade 0. All subjects included in this clinical trial were negative for BMP-2 antibodies in the BMP-2 immune response test during the clinical trial period.

## Discussion

The present study investigated the efficacy and safety of rhBMP-2/β-TCP at 12 weeks after ARP using CT evaluation and compared it with that of β-TCP. The addition of rhBMP-2 to β-TCP provided a higher alveolar bone preservation effect for both alveolar bone height and width than β-TCP, with a safety profile similar to that of β-TCP.

β-TCP, which was used as a bone substitute and a BMP-2 carrier in our study, has already been used for ARP [24–26]. Horowitz et al. [24] grafted a pure-phase β-TCP to preserve the volume of the alveolar ridge after tooth extraction with a barrier membrane and evaluated the width of the extraction socket 6 months after surgery. In their study, the alveolar bone width was preserved to 91% of the preoperative width. Several investigators have reported new β-TCP-based materials to enhance intraoperative manipulation and stability in the extraction socket. In the study by Leventis et al. [25], in situ hardening β-TCP coated with poly lactic-co-glycolic acid (PLGA) was grafted onto the extraction socket for ridge preservation. Coating with PLGA can improve the manipulation of graft materials and provide in situ hardening and stable maintenance of the graft material in the socket. The histological evaluation at 4 months after treatment found new bone regeneration of 24.4 ± 7.7%. Saito et al. [27] evaluated the efficacy of PLGA-coated β-TCP for ARP purposes compared to freeze-dried bone allografts and reported comparable outcomes in maintaining alveolar bone dimensions. Combining β-TCP with collagen has been proposed to promote bone regeneration and provide better manipulation capability than TCP alone [6, 26]. Brkovic et al. [6] performed ARP using a bone substitute in which β-TCP and type I collagen were combined, and Takahashi et al. [26] investigated the efficacy of β-TCP/collagen composite for ridge preservation in an experimental study in dogs. In the study by Takahashi et al. [26], β-TCP/collagen composite replacement by new bone formation at 8 weeks after treatment with similar ridge preservation to β-TCP. In our clinical trial, β-TCP was used as an injectable type of β-TCP, which consisted mainly of β-TCP and poloxamer hydrogels [28]. Poloxamer is known to have thermoreversible properties, which can allow the formation of gel-like composites at room temperature to facilitate solubilization of poorly soluble drugs and form a gel state at body temperature [29, 30]. The characteristics of injectable substitutes could help investigators to easily apply bone substitutes to the extraction socket. The ridge preservation effect of β-TCP on alveolar bone height was 83.44% in alveolar bone height, and that in alveolar bone width was 92.74% at 75% ESL, 82.27% at 50% ESL, and 55.11% at 25% ESL in alveolar bone width, compared with the alveolar ridge immediately after extraction.

Previously, the use of rhBMP for ARP after tooth extraction has been reported. Fiorellini et al. [22] assessed the efficacy of rhBMP-2 delivered on an ACS for bone induction and reported enhanced bone regeneration in combination with rhBMP-2. In their study, the patients with 1.50 mg/mL rhBMP-2/ACS had significantly greater bone augmentation than the no-treatment group. While the no-treatment group exhibited a 1.17-mm reduction in alveolar bone height and a 1.62-mm increase in width change at 50% ESL, the 1.50 mg/mL rhBMP-2/ACS group exhibited a 0.02-mm reduction in alveolar bone height and a 3.97-mm increase in width change at 50% ESL. In another randomized clinical trial by Jo et al. [10], in which the efficacy of two rhBMP-2 delivery systems for ARP was evaluated, the dose of rhBMP-2 was applied by 1.5 mg/mL for ACS and 1.0 mg/mL for β-TCP /HA. In our study, low-dose rhBMP-2 (0.5 mg/site) was applied to β-TCP for the test group, and the efficacy and safety of the graft material were assessed.

A previous clinical trial has shown the efficacy of rhBMP-2 on bone regeneration in ARP using an alloplastic bone graft [20]. In this clinical trial, Escherichia coli-derived rhBMP-2 (ErhBMP-2)-coated β-TCP/HA was grafted into the extraction socket of premolars or molars for ridge preservation, and the remaining alveolar ridge was assessed using CT data at 3 months after treatment. In their study, changes in alveolar bone height were significantly less in the ErhBMP-2 + β-TCP/HA group (− 0.06 mm) than in the β-TCP/HA group (− 1.09 mm). Concerning alveolar bone width, greater bone regeneration was observed in the ErhBMP-2 β-TCP/HA group (1.28 mm, 1.24 mm, and 1.86 mm at 25%, 50%, and 75% ESL, respectively) than in the β-TCP/HA group (0.01 mm, 0.54 mm, and 1.41 mm at 25%, 50%, and 75% ESL, respectively). The effect of rhBMP-2 suggested by these results is consistent with our results. In our study, the rhBMP-2/β-TCP group exhibited significantly less bone resorption in height than the β-TCP group (rhBMP-2/β-TCP, − 0.50 mm; β-TCP, − 1.42 mm). The rhBMP-2/β-TCP group also exhibited a significantly greater ridge preservation effect in width than the β-TCP group (− 0.93 mm, − 0.19 mm and − 0.23 mm for rhBMP-2/β-TCP group and − 3.96 mm, − 1.69 mm, and − 0.70 mm for the β-TCP group at 25%, 50%, and 75% ESL, respectively). However, in contrast to the study by Huh et al. [20] in which the alveolar bone width increased in all groups 3 months after treatment, both groups in our study exhibited a significant decrease in alveolar bone width. One explanation for this difference may be that the study design and measurement methods in the two clinical trials were different, and the position of the teeth included in the two clinical trials may also be one of the possible reasons.

Regarding safety, there were no severe adverse events related to the graft material or procedures. Although one of 13 adverse events exhibited a relationship with the graft material, the adverse event showed mild severity and resolved completely with medication. In the healing status assessment and laboratory examination, no clinically significant changes or findings were found along with a negative response to BMP-2 antibodies in the immune response test. These results suggest that graft materials and protocols can be safely utilized for ARP.

Although there are few randomized clinical trials that applied rhBMP-2 to alveolar ridge preservation, grafting of carriers such as ACS, β-TCP, and β-TCP/HA together with rhBMP-2 has been shown to have a higher alveolar ridge preservation effect than grafting with carrier alone [10, 20, 22]. In particular, the effect of preserving the alveolar ridge was greater in the alveolar bone width than in the alveolar bone height. Considering maintaining sufficient volume of alveolar ridge through ARP will allow to place implants at the optimal position with favorable angulation and enable functional and esthetic prosthesis, the clinical application of rhBMP-2 is worth considering. Although our study also revealed the alveolar ridge preservation effect of rhBMP-2 at a low-dose concentration of rhBMP-2, further studies are needed to find out the safe and optimal concentration of rhBMP-2 for alveolar ridge preservation. Based on the results of the present study, the bone-forming effect of rhBMP-2/β-TCP in other types of osseous defect will be evaluated in further studies, such as alveolar bone or maxillary sinus floor augmentation, bone gap in jaw fracture, and orthognathic surgery.

There are several limitations to this study. The first is the lack of histomorphometric analysis. Although CT evaluation may be a non-invasive method, early bone formation where the bone density is relatively low to examine in the CT evaluation can be assessed precisely using histomorphometric analysis. Second, except for the third molar, the position of the teeth was not applied to the selection criteria. Each tooth has a different thickness of alveolar bone on the buccal/labial and lingual/palatal sides, depending on the tooth position. In particular, the labial bone in the anterior region is very thin. However, the thickness of the buccal bone in the posterior region may be relatively thick. Therefore, to obtain more consistent and reliable results, it may be necessary to be further subdivided according to the position of the teeth and to consider the thickness of the cortical bone of the extraction socket and the width of the alveolar ridge.

In conclusion, this clinical trial showed that rhBMP-2/β-TCP provided a better ARP effect for both the height and width of the alveolar bone with a safety profile similar to that of β-TCP. Therefore, rhBMP-2/β-TCP is determined to be a safe graft material that provides a high alveolar bone preservation effect in patients undergoing dental extraction.

## Data Availability

The datasets used and analyzed during the current study are available from the corresponding author on reasonable request.
